# The Role of Different Afferent Systems in the Modulation of the Otolith-Ocular Reflex After Long-Term Space Flights

**DOI:** 10.3389/fphys.2022.743855

**Published:** 2022-03-14

**Authors:** Dmitrii O. Glukhikh, Ivan A. Naumov, Catho Schoenmaekers, Ludmila N. Kornilova, Floris L. Wuyts

**Affiliations:** ^1^Laboratory of Vestibular Physiology, Russian Federation State Scientific Center – Institute of Biomedical Problems of the Russian Academy of Sciences (SSC RF – IBMP RAS), Moscow, Russia; ^2^Lab for Equilibrium Investigations and Aerospace (LEIA), University of Antwerp, Antwerp, Belgium

**Keywords:** otolith-ocular reflex, ocular counter-rolling, afferent systems, spaceflight, centrifugation

## Abstract

**Background:**

The vestibular (otolith) function is highly suppressed during space flight (SF) and the study of these changes is very important for the safety of the space crew during SF missions. The vestibular function (particularly, otolith-ocular reflex–OOcR) in clinical and space medicine is studied using different methodologies. However, different methods and methodologies can influence the outcome results.

**Objective:**

The current study addresses the question of whether the OOcR results obtained by different methods are different, and what the role is of the different afferent systems in the modulation of the OOcR.

**Methods:**

A total of 25 Russian cosmonauts voluntarily took part in our study. They are crewmembers of long duration space missions on the International Space Station (ISS). Cosmonauts were examined in pre- and post-flight “Sensory Adaptation” and “Gaze Spin” experiments, twice before (preflight) and three times after SF (post-flight). We used two different video oculography (VOG) systems for the recording of the OOcR obtained in each experiment.

**Results:**

Comparison of the two VOG systems didn’t result into significant and systematic differences in the OOcR measurements. Analysis of the static torsion otolith–ocular reflex (OOR), static torsion otolith–cervical–ocular reflex (OCOR) and static torsion otolith–ocular reflex during eccentric centrifugation (OOREC) shows that the OOREC results in a lower OOcR response compared to the OOR and OCOR (before flight and late post-flight). However, all OOcRs were significantly decreased in all cosmonauts early post-flight.

**Conclusion:**

Analysis of the results of ocular counter rolling (OCR) obtained by different methods (OOR, OCOR, and OOREC) showed that different afferent systems (tactile-proprioception, neck-cervical, visual and vestibular afferent input) have an impact on the OOcR.

## Introduction

Long-term weightlessness during SF is a unique way to modify the input signals from the otoliths, which allows the identification of the character and dynamics of vestibular function changes (VF). These modifications are accompanied by the development of space adaptation syndrome and space motion sickness. Changes can occur during flight as well as during the initial period after landing on Earth (Kornilova et al., 2017).

A retrospective analysis of the otolith-ocular reflex (Miller and Graybiel, 1971; Homick and Reschke, 1977; Baumgarten et al., 1982; Diamond and Markham, 1983, 1998; Oman et al., 1986; Reschke et al., 1986; Watt, 1987; Moore et al., 1991; Kornilova, 1997; Young and Sinha, 1998), obtained by different researchers before and after spaceflight, showed contradictory results. Some of the researchers found significant changes in OCR after spaceflight, while others (Clément and colleagues) found no significant change in OCR after flight compared with before flight in 18 astronauts tested after (short term) shuttle missions (Clément et al., 2007). A possible reason for this could be the duration of the shuttle missions, <2 week, while the data collected in other studies cover astronauts and cosmonauts who spend 6 months in space. Another difference, however, may be due to the difference in vestibular stimulation between static tilt and centrifugation (Moore et al., 2005).

A recent study (Hallgren et al., 2016) has shown a decreased OCR reflex in the 25 astronauts after long term spaceflight. Thus, this confirmed the previously observed significant changes in OCR after spaceflight. However, when compared with previously published data, new questions arose: different dynamics of OCR changes after spaceflight and the absence of pronounced atypical changes (inversion or complete absence of the otolith reflex). Data comparison was also complicated by different data samples (different astronauts and cosmonauts participated in various studies). Moreover, a comparative analysis of the otolith reflex under conditions of a real and simulated (“dry” horizontal immersion or head-down tilt bed rest) weightlessness (Naumov et al., 2021) demonstrated the influence of non-vestibular afferentation on the intensity of the otolith-ocular reflex and its dependence on other sensory inputs. While not directly affecting the vestibular input, prolonged dry immersion and bedrest nevertheless led to changes in the otolith-ocular reflex similar to changes observed post-flight.

Thereby, the inconsistency in OCR studies could be due to a different flight duration, number of flights, individual adaptation mechanisms and as well as characteristics of the onboard activities [countermeasures, dynamic flight operations (EVA)] during spaceflight. Additionally, different methodological approaches, instructions and techniques adopted by the scientists who tested the cosmonauts are hypothesized as contributing factors to the inconsistencies regarding the observed otolith responses.

In this study, we tried to take a more rigorous approach. The main aim of the current study was to conduct a comparative analysis of the OOcR obtained by different methodologies in the same cosmonauts before and after long duration SF and to evaluate the role of different afferent systems in the modulation of the otolith-ocular reflex. An additional goal was to conduct a comparative analysis of the OOcR obtained by different methodologies based on measurements of an artificial eye.

## Materials and Methods

The 25 Russian cosmonauts (37–59 years old, average age of 46) who enrolled in this study participated in the International Space Station (ISS) increments 29 till 46. All crewmembers experienced microgravity aboard the ISS, with a duration between 124 and 340 days. Cosmonauts were examined in pre- and post-flight clinical and physiological examinations and during two space experiments: “Sensory Adaptation” (SA) and “Gaze Spin” (GS). All cosmonauts took part in both experiments for assessment of the otolith–ocular reflex (OOR)/otolith–cervical–ocular reflex (OCOR) (SA), and otolith–ocular reflex during eccentric centrifugation (OOREC) (GS). All tests were performed in the Yuri Gagarin Cosmonaut Training Center (GCTC) in Star City near Moscow (Russian Federation). Two preflight measurements were conducted 1–2 months before spaceflight and consisted of 2–3 baseline recordings (called Baseline Data Collection–BDC). The2–3 post-flight measurements were conducted in the first 2 weeks after landing. These post-flight time points are denoted as “early” when the cosmonaut was tested on the 2–3 days after landing (R + 2/3), “mid” when tested between the 4 and 5 days after landing (R + 4/5), and “late” when tested the 9–12 days after landing (R + 9/12). It was impossible to test all cosmonauts on the same day, due to medical and organizational limitations.

All participants gave their written informed consent prior to their participation.

The state of the vestibular function during SA and GS experiments was recorded using video oculography (VOG) method.

During the SA experiments, described earlier by prof. L. Kornilova and her team (Kornilova et al., 2007a,b, 2011b,^2012^), the otolith mediated ocular reflex, representing the vestibular function in cosmonauts, was evaluated using two different methodologies (Kornilova et al., 2007c).

### Static Torsion Otolith–Ocular Reflex and Static Torsion Otolith–Cervical–Ocular Reflex

The OOR Was Assessed as an Amplitude of Compensatory Ocular Counter-Rolling When the Body (With a Fixed Head and Straight Neck) Was Tilted Upon a Verbal Command to the Right and Left Side With an Amplitude of 30°

**FIGURE 1:**
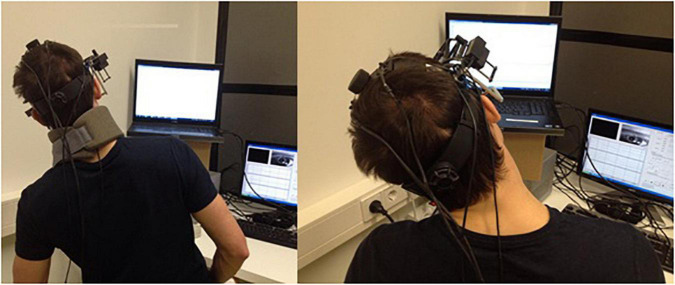
Visual representation on how the OOR and OCOR were conducted. Right, whole body tilt with restricted neck/head movement (OOR). Left, head tilt (OCOR).

The OCOR was assessed as an amplitude of compensatory ocular counter-rolling (OCR) when the head was 30° tilted, upon a verbal command, to the right and left shoulder. In order to remove the dynamic impact on the static reflex, the subject’s head or body was kept tilted in each position for at least 16 s. The angle of tilt was recorded, and the position was manually monitored and controlled by the instructor using an inclinometer (Figure 1).

The measurements of the OCOR and OOR were performed in a sitting position and in a darkened room after a 2-min dark adaptation. The OOR measurements were performed with a fixed head using a neck collar.

During both measurements, the horizontal, vertical, and torsional eye movements were recorded using the Chronos Vision Eye Tracking Device (ETD) (Berlin, Germany). The VOG helmet was equipped with high-frequency infrared video cameras with a recording frequency of 200 frames per second. The range of recording of the horizontal and vertical eye movements was up to 55 and 35°, respectively. VOG recordings were processed using the ETD Iris Tracker built-in software; the accuracy of recognition of the eyes position in all planes was <0.05°.

The VOG calibration was performed using the so-called “5-point calibration,” the gaze fixation and tracking of the sequence of saccadic movements of a visual target (with a size of about 1°) by 10° to the left/right, upward/downward, and to the center. Subsequently, horizontal and vertical eye movements were analyzed in the VOG recordings by recognizing the pupil center using the Hough transformation, a feature extraction technique used in image analysis. Using artificial neural networks and cross-correlation analysis made it possible to detect the torsional displacement of the iris segment (Clarke et al., 2002).

The outcome measures of the static tests were defined as the gain of the OOR and OCOR (the ratio between the angles of OCR and tilts of the head/body) denoted, respectively, as gOCOR and gOOR.

### Static Torsion Otolith–Ocular Reflex During Eccentric Centrifugation

During the Gaze Spin experiment the same otolith mediated ocular reflex was evaluated during rotation on a short radius off-axis centrifuge resulting in the third otolith-ocular reflex measurement–OOREC.

**FIGURE 2:**
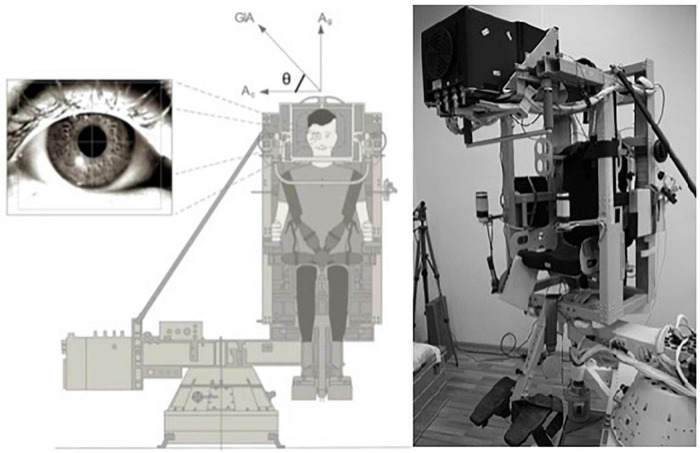
The Visual and Vestibular Investigation System (VVIS) and perception of GIA for evaluation of the otolith mediated ocular reflex during centrifugation (OOREC).

**FIGURE 3:**
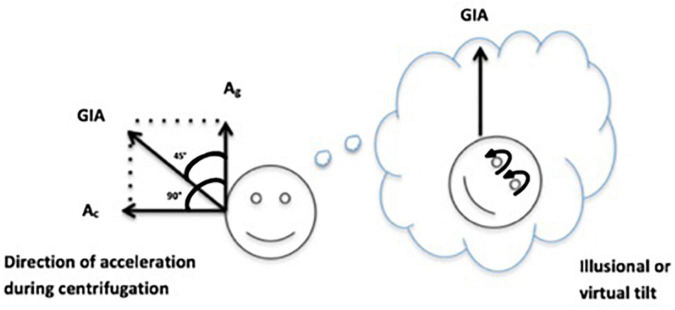
Visual representation on how the subject perceives GIA and the ocular counter roll (OCR).

The OOREC was evaluated as an amplitude of compensatory OCR when the test subject was rotated on the VVIS (Visual and Vestibular Investigation System) chair–a small off-axis centrifuge built by the European Space Agency (ESA) for the Neurolab shuttle mission (Buckey and Homick, 2003). The test subject was securely fixed in the chair and head movements were restricted. The entire room was darkened to avoid visual motion feedback during rotation. The centrifuge (Figure 2) allowed earth vertical rotation with the subject placed on a fixed distance of 0.5 m from the axis of rotation. In front of the test subject, a screen was placed on which visual targets were presented during parts of the experiment. After a baseline recording, the cosmonaut was subjected to 1 g for 5 min in a counterclockwise (CCW) direction and subsequently 5 min in a clockwise (CW) direction. In between rotations, the subject’s seat orientation was rotated 180°. As a result, the subject was always facing the direction of motion, i.e., right-ear out (REO) during CCW rotation and left-ear out (LEO) during CW rotation. The maximum velocity of 254°/s was chosen to obtain an outward centripetal acceleration of 1 g and an upward gravitational acceleration of 1 g, such a shear force constitutes a virtual sideways tilt of 45°. The resultant of accelerations acting upon the head and body is called the gravito-inertial acceleration (GIA) depicted on Figure 3.

The OCR, torsional movements of the eyes, was recorded using a three-dimensional infrared AUREA (Antwerp University Research center for Equilibrium and Aerospace) VOG system built by Hamish MacDougall (Sydney University) and used in previous studies for OCR recordings (Hallgren et al., 2015, 2016). As a second step we analyzed the recorded OCR eye movements, using a visual programming language (custom made in LabView by Hamish MacDougall–National Instruments–11500 N Mopac Expwy, Austin, TX, United States). Subsequently, the gain of the OOREC was calculated as the ratio between the degree of the OCR and the tilt of 45° induced by GIA (gOOREC).

During off-axis centrifugation, the net linear acceleration stimulating the otoliths is the (vector) sum of the centripetal and gravitational acceleration, GIA (see vectors in the Figure 3). During centrifugation GIA is interpreted as the true vertical, with the consequence that the subject experiences a sensation of tilt.

Since we used two different VOG systems for the analysis of the otolith reflex, we compared both systems to rule out systematic differences. Hereto, we used an artificial eye and a specific calibration system holding the goggles in place. To estimate the accuracy of both VOG systems, used to measure ocular counter rolling (OCR), we used a 5-point calibration and 8 torsional tests where the artificial eye was rotated over 5°CW and CCW.

### Analyses

The quantitative and comparative analysis of parameters of our study were performed by use of parametric and non-parametric statistical methods as well as correlation analyses. Mean, variance, variation range, and coefficient of variation were evaluated for each parameter. In all cases of examining our statistical hypotheses [the normality of distributions, homogeneity (equality) of variances, significance of differences, etc.], the critical level of significance α was 0.05. The hypotheses about the statistically significant differences between preflight and post-flight values were examined using a repeated measures ANOVA.

The normality of distributions was examined using the Kolmogorov–Smirnov/Lilliefors test, and the homogeneity of variances was examined using the Levene’ test. Statistical analyses were made in excel, Matlab and SPSS Statistics.

## Results

### Comparison of the Video Oculography Systems

Based on repeated measurements of each of the VOG system (AUREA, VOG, and ETD), it was possible to obtain sufficient data for analyzing the torsional movement of the artificial eye (Table 1). There was no statistically significant difference between both systems (Mann Whitney *U*-test). Furthermore, the difference [or delta Δ (%)] was within 5% in average value of these measurements for each system and CW and CCW eye movements.

**TABLE 1:** Comparison of ETD and AUREA VOG systems.

		Left tilt (degrees°)	Right tilt (degrees°)
AUREA	Average	–4.88	5.19
	Standard deviation	0.15	0.16
ETD	Average	–4.68	4.94
	StandarddDeviation	0.23	0.11
	Δ (%)	**4.34**	**4.93**

*Bold values indicates % of error (StDev).*

Therefore, we can conclude that measurements of the otolith reflex obtained by different VOG-systems are unbiased and independent from the type of used system.

### Otolith–Ocular Reflex, Otolith–Cervical–Ocular Reflex, Otolith–Ocular Reflex During Eccentric Centrifugation

According to the processed results obtained in the Laboratory of Vestibular Physiology of the Institute of Biomedical Problems (IBMP) (Kornilova L.N., Naumov I.À., Glukhikh D.O., Ekimovskiy G.À.), there was no statistically significant difference between the OOR and OCOR measurements within the same group of cosmonauts (Naumov et al., 2021). However, studies of the OOR and OCOR pre- and post-flight measurements have shown a significant reduction in the tonic (static) components of the vestibulo-ocular responses during post-flight readaptation. In some cases (22%), a complete abolishing or inversion of the OCR was observed (Naumov et al., 2015; Kornilova et al., 2017).

Preflight, the amplitude of compensatory OCR was within the physiological range of 4–8°. The reflex was symmetrical, except for one cosmonaut who had an OCR of 4° in tilting the head to the left side, but had an OCR of −7°to the right side.

Repeated measures ANOVA revealed a statistically significant time effect for all cosmonauts. A significant decrease of the OOR/OCOR was observed 2–3 days after their return (R + 2/3), compared to BDC (preflight). The decrease was still significant, but less, 4–5 days after their return (R + 4/5) compared to BDC. At day 9–12 after their return (R + 9/12), no significant difference was observed any more, compared to BDC.

**FIGURE 4:**
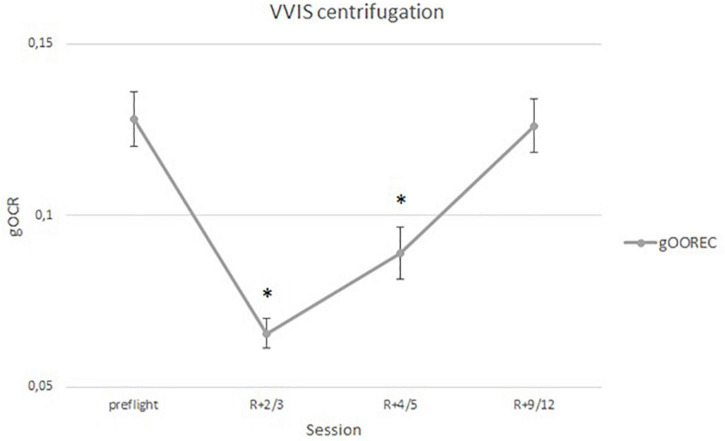
The gOOREC (mean ± SD) for the 25 astronauts before and after SF, averaged over the two directions of rotation and both eyes (^∗^–significant difference from BDC, *p* < 0.05).

Analysis of the vestibulo-ocular reflex during centrifugation on Figure 4 shows the mean alteration of OOREC for the same 25 cosmonauts, averaged over both directions of rotation and both eyes (Hallgren et al., 2016). The figure displays the OOREC for the preflight session [0.13 gOOREC (6.4° ± SE)], as well as the three post-flight sessions of the experiment. For the majority of the analyses, the focus was on the contrast between the OOREC preflight and early post-flight measurements. Most of the cosmonauts were tested on day three and/or on day five after return. At approximately 5 days after return, the otolith function starts to re-adapt and at day nine we see that most of them are fully re-adapted to Earth’s gravitational level, 1 g.

### Comparison of Reflexes Obtained by Different Methods

**FIGURE 5:**
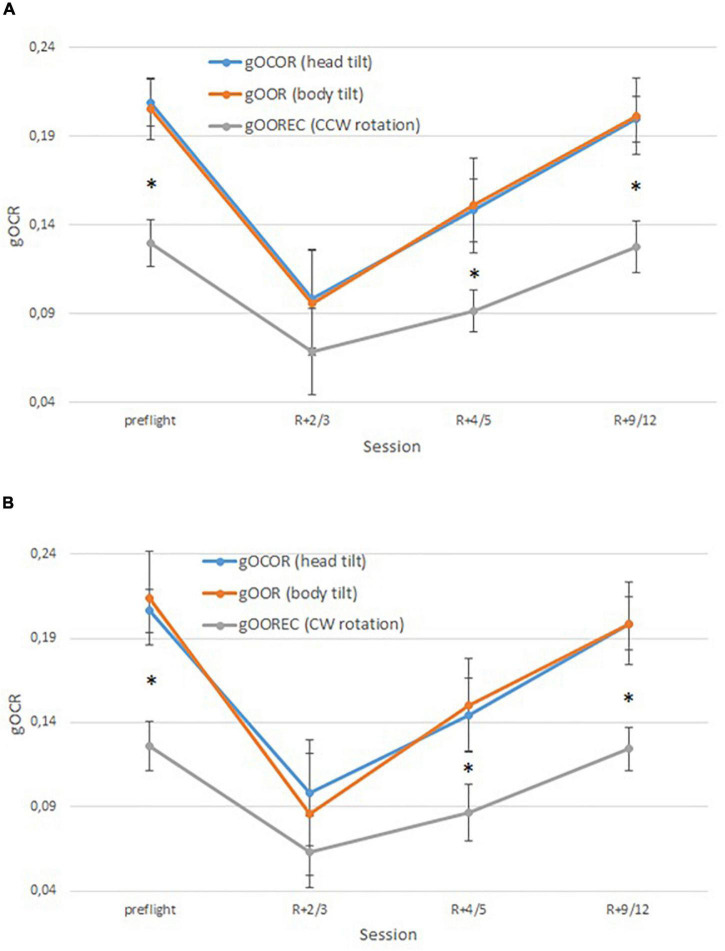
Comparison of gOOR/gOCOR/gOOREC (**A**–tilt body/head to the right/CCW rotation, **B**- tilt body/head to the left/CW rotation, ^∗^–significant difference between gOCOR/gOOR and gOOREC, *p* < 0.05).

Clockwise rotation, inducing a subjective tilt to the left, was compared with the static head and body tilt to the left. CCW rotation was compared with the static head and body tilt to the right. For all 25 cosmonauts, we obtained statistically significant differences between OCR reflexes obtained in static head/body tilts compared to rotation on the VVIS chair on BDCs, R + 4/5 and R + 9/12. No difference was observed early post-flight (R + 2/3). Figure 5 represents the OCR data for the three different methodologies (static head tilt, static body tilt, centrifugation) for all cosmonauts and for both centrifugation directions.

## Discussion

It is well known that SF, presented as a form of sensory stimulus and through the central mechanism of reinterpretation, requires changes of the established response patterns which are constant in Earth’s gravitational condition. Evidence of sensory reinterpretation is appearing as post-flight changes in static vestibular-cervical-ocular responses (Dai et al., 1994, 1998; Clement and Reschke, 1996; Clément et al., 2001).

The Russian space experiment SA, which induces a static head/body tilt, has shown a significant decrease of the amplitude or even inversion and absence of the compensatory ocular counter-roll during post-flight measurements (Naumov et al., 2015; Kornilova et al., 2017). The observed changes of the otolith-ocular reflex and otolith-cervical-ocular reflex (OOR/OCOR) seems to be determined by reflex mechanisms (as confirmed by a decreased but still present OOR/OCOR during a static head tilt due to cervical proprioceptive afferentiation), and by the central deafferentation of the vestibular afferent signal in early readaptation to the gravitational field of Earth (as confirmed by inversion or absence of the OOR/OCOR).

However, in four astronauts of the Neurolab (STS-90) expedition the amplitude of the otolith-ocular reflex in-flight and post-flight was almost unchanged (Moore et al., 2001), probably due to the in-flight centrifugation. Therefore, an intermittent exposure to artificial gravity could prevent changes of the otolith-ocular reflex in microgravity.

Post-flight data suggests that adaptation to microgravity is accompanied by a central, deep and prolonged suppression of the OCOR. Recovery, after return to Earth, takes some time during which there is a readaptation of the otolith function to the Earth’s gravitational level of 1 g take place (Kornilova et al., 2012, 2017). It is known that in these recovery processes, the nodulus and uvula of the cerebellum play an important role (Cohen et al., 2002, 2019).

The phenomenon of the decreased or absent static torsion OCR observed in- and post-flight is consistent with the results of histological studies in monkeys and rats exposed to microgravity (Moore et al., 2003). They showed that morphological features of hypofunction of the utriculus receptor cells reduces the afferent input to the vestibular nuclei, which can result in an impairment of vestibular impulses to the cerebellum flocculus.

Given that no statistically significant differences were found between the used VOG systems, we can attribute the observed difference between the static head and body tilts and dynamic centrifugation test purely to physiological processes. We did not find statistically significant differences between the OOR and OCOR measurement methodologies. It can be the cause of different afferent systems (tactile-proprioception, neck-cervical, visual and vestibular afferent input) that were included in the process of the reflexes. The difference between the two tests is that there is a contribution of neck afferentation during the OCOR test, however, during the OOR test there is a contributing afferentation from low tonic back muscles condition on the back, after long-term exposure in weightlessness. Previous research has shown that the ocular counter roll induced by head tilt does not differ from whole body tilt (Zingler et al., 2006). Results of assessment of changes in vestibular function and, particularly in otolith function after long-term space flight, obtained by Russian researchers (Kornilova, 1997, 2001; Clarke et al., 2000; Kornilova et al., 2002, 2007a,b,^2017^; Hallgren et al., 2015, 2016; Naumov et al., 2015). It was found that there is a decrease in OCR early after return from space, and that this reflex regains the preflight amplitudes approximately 1–2 weeks after return in the normal gravitational environment of Earth.

Thus, the only remaining novel observation in our experiment to be explained is the preflight difference between centrifugation induced OCR compared to a static head/body tilt, as well as why this difference disappears early post-flight. One can hypothesize that the OCR induced by centrifugation is a function of several components. The most prominent one is the shear force, induced by GIA, on the otoliths that generates an OCR. However, there is a difference between the left and right otolith system since on average the inner otolith is located at a distance of 46 cm from the center of rotation and the outer at 54 cm (Nowé et al., 2003). This difference in distance results in a 17.4% difference in g-force. This difference between both otolith systems may cause an inhibition in OCR output, since it actually consists of an intra-vestibular conflict (Wetzig et al., 1990). It can be hypothesized that conflicts within the central nervous system can lead to inhibition of reflexes, to limit the conflict. This can even take place at the cortical level, as shown in a parabolic flight study (Demertzi et al., 2015; Van Ombergen et al., 2017). Similar inhibition of VOR gains during rotation to the healthy side are observed in the early phase of a vestibular neuritis in dizzy patients to limit the degree of asymmetry (Cohen et al., 2017). This could be a first reason why the OCR is reduced during centrifugation compared to a static body or head tilt.

The second component is an OCR generated by the horizontal semicircular canals as demonstrated by Buytaert et al. (2010). This torsional component is seldom recorded since usually the horizontal nystagmus is much more intense compared to the torsional component. However, when the subject focusses on a target, at specific moments during centrifugation, the canal induced torsional component is present, even 70 s after the acceleration phase when the OCR in opposite direction of the tilt was measured. The time constant of this canal driven OCR is of several hundreds of seconds (Buytaert et al., 2010).

The third component is the proprioceptive input of the chair. When centrifuged in the VVIS chair, the subjects are experiencing a sideway force of 1.6 G at the level of their shoulders and thighs. Despite the use of foam to soften the contact, this proprioceptive input is very prominent and the afferentation pathways add to the otolith input in such a way that the pure otolith reflex is modulated and inhibited. This results in a smaller ocular counter roll that exists prior to space flight as well as a week or longer after landing. Additionally, the sideways push can also give the subject the illusion of undergoing a sideways translation, rather than a tilt, which could also influence the otolith mediated output. This strong proprioceptive input can have a considerable impact on the inhibition of the OCR.

Comparing experiments, one can see a difference in vestibular stimulation between static tilt (which elicits response from the vertical semicircular canals) and centrifugation (which does not). During centrifugation even when fully secured in chair a subject unconsciously strains his body. He is also pressed into the chair during rotation–thus, there is additional tactile-proprioceptive afferentation as well.

However, in the early phase after landing, there was no statistically significant difference observed between both modalities. This could be due to the fact that during space flight the tactile-proprioceptive input was deafferentation to a certain extent (Reschke et al., 1998; Kornilova and Kozlovskaya, 2003), because of the limited tactile-proprioceptive input in prolonged microgravity conditions. Several days after landing, the tactile-proprioceptive afferentation recovers, thereby reducing the ocular counter roll induced by centrifugation. This leads to the observed OCR difference between the static head/body tilt vs. centrifugation. Additionally, the otolith system itself is deprived of afferent information during the extended period of space flight, leading to the known OCR decrease early after landing (Clarke et al., 1993, 2002; Clarke and Kornilova, 2007; Hallgren et al., 2016). The inhibitory influence of the canals may very well be hampered, since the intra-vestibular conflict could lead to a central suppression in the gains of the different systems, similar to the one observed during vestibular neuritis. More central inhibition of the canal input will lead to less inhibition of the OCR. As the result, the reduction of the OCR early post-flight is therefore similar to the one observed during a static body and head tilt.

## Conclusion

During our study we observed several findings that can help us try to understand the mechanisms of otolith-driven reflexes and continue studying the vestibular system with a new point of view on the process:

- Analysis of the VOG systems, based on different types of measurements systems which were included in this study showed no statistically significant difference in the otolith reflex;
- After a long-term exposure to microgravity, the otolith system among returning cosmonauts was highly affected;
- The OOR/OCOR/OOREC reflex was significantly decreased in the 25 cosmonauts who took part in this study;
- Inversion or full absence of OOR/OCOR during the initial readaptation period after long-term SF is due to central deafferentation (“rejection”) of the changed vestibular signal in weightlessness.

Late post-flight, 9–12 days after return (R + 9/12), the OOR/OCOR/OOREC was back at preflight values, indicating a full recovery or readaptation of the otolith system;

- Analysis of the OOcR, obtained by different methods, has shown the dependence from the involved tests of different afferent systems. The difference in OOR/OCOR and OOREC can be explained by afferentation from shoulder proprioceptors subjected to 1.6 G centripetal acceleration. The difference in tactile-proprioceptive input can change the functioning of the vestibular system and particularly the otolith reflex. This has also been observed in earlier studies, in space model experiments such as the Dry Immersion and BedRest studies (Kornilova et al., 2010, 2011a,^2013^);
- The here performed comparison in the same subjects, pre- and post-flight, shows that the method applied to evaluate the otolith mediated reflex has an influence on the outcome results. Higher gains were observed with static sideway tilts compared to off axis centrifugation, except early post-flight (R + 2/3). Possibly due to additional afferent systems, such as proprioception, that modify the OOcR. These findings may explain several earlier found differences in the literature between different used methods for assessing otolith mediated reflexes.

## Data Availability Statement

The original contributions presented in the study are included in the article/supplementary material, further inquiries can be directed to the corresponding author.

## Ethics Statement

The studies involving human participants were reviewed and approved by the Bioethic committee of the SSC RF – IBMP RAS and Human Research Multilateral Review Board (HRMRB). The patients/participants provided their written informed consent to participate in this study.

## Author Contributions

LK and FW designed the research. DG wrote the manuscript. DG, IN, FW, and CS performed experiments and analyzed data. LK, FW, DG, and IN discussed the results. All authors drafted the work critically and approved the final version, and agreed to be accountable for all aspects of the work in ensuring that questions related to the accuracy or integrity of any part of the work are appropriately investigated and resolved.

## Conflict of Interest

The authors declare that the research was conducted in the absence of any commercial or financial relationships that could be construed as a potential conflict of interest.

## Publisher’s Note

All claims expressed in this article are solely those of the authors and do not necessarily represent those of their affiliated organizations, or those of the publisher, the editors and the reviewers. Any product that may be evaluated in this article, or claim that may be made by its manufacturer, is not guaranteed or endorsed by the publisher.
